# Pre- and postoperative alcohol consumption in breast cancer patients: impact on early events

**DOI:** 10.1186/2193-1801-3-261

**Published:** 2014-05-22

**Authors:** Maria Simonsson, Andrea Markkula, Pär-Ola Bendahl, Carsten Rose, Christian Ingvar, Helena Jernström

**Affiliations:** Department of Clinical Sciences, Division of Oncology and Pathology, Barngatan 2B, SE-22185 Lund, Sweden; CREATE Health and Department of Immunotechnology, Lund University, Medicon Village, Building 406, Lund, Sweden; Division of Surgery, Clinical Sciences, Lund, Lund University, Lund, Sweden; Department of Surgery, Skåne University Hospital, Lund, Sweden

**Keywords:** Breast cancer, Alcohol consumption, Early breast cancer events, Early distant metastases, Death

## Abstract

**Purpose:**

To investigate the association between pre- and postoperative alcohol consumption and risk for early breast cancer events, since the association between alcohol consumption and prognosis in breast cancer patients is unclear.

**Methods:**

Alcohol consumption was recorded for 934 primary breast cancer patients who underwent breast cancer surgery in Lund, Sweden, between 2002 and 2011 and were followed until December 31^st^ 2012. Clinical data were obtained from medical records and population registries. Pre- and postoperative alcohol consumption was analyzed in relation to risk for early events.

**Results:**

Median follow-up time was 3.03 years and 100 breast cancer events, 65 distant metastases, and 76 deaths occurred. Compared to no consumption, any preoperative alcohol consumption was weakly associated with lower risk for early events, adjusted HR 0.69 (0.45-1.04), distant metastases, 0.60 (0.36-1.00) and death, 0.62 (0.38-1.01). In the 572 patients without axillary lymph node involvement, any alcohol consumption was not associated with risk for early events. However, in the 360 patients with axillary lymph node involvement, preoperative alcohol consumption was associated with lower risk for early events (adjusted HR 0.43 0.24-0.77; *P*_interaction_ = 0.01).

**Conclusion:**

Pre- and postoperative alcohol consumption was weakly associated with lower risk for early breast cancer events. The data does not support recommending that all breast cancer patients abstain from low to moderate alcohol consumption.

## Background

The risk for the development of breast cancer in relation to alcohol consumption has been thoroughly studied. Alcohol intake, both light and heavy drinking, is now identified as a risk factor for pre- and postmenopausal breast cancer (Singletary and Gapstur 2001;Hamajima et al. 2002;Pelucchi et al. 2011;Chen et al. 2011;Seitz et al. 2012). If and how alcohol consumption is associated with survival and disease recurrence is, however, not yet completely understood. The results from previous studies are inconsistent. The majority of studies report no association between alcohol consumption and overall survival (Holmes et al. 1999;Dal Maso et al. 2008;Hellmann et al. 2010;Kwan et al. 2010;Ali et al. 2014) or breast cancer-specific survival (Dal Maso et al. 2008;Harris et al. 2012;Holm et al. 2013). Some studies report a protective effect from alcohol consumption on overall survival (Harris et al. 2012;Reding et al. 2008;Barnett et al. 2008;Flatt et al. 2010) and breast cancer-specific survival (Ali et al. 2014;Reding et al. 2008;Flatt et al. 2010;Newcomb et al. 2013), while other studies report higher breast cancer-specific mortality (Kwan et al. 2010;Allemani et al. 2011;Vrieling et al. 2012). Five studies have investigated the association between alcohol consumption and recurrence of breast cancer (Kwan et al. 2010;Holm et al. 2013;Flatt et al. 2010;Vrieling et al. 2012;Kwan et al. 2013), and two of these report an increased risk of breast cancer recurrence (Kwan et al. 2010;Holm et al. 2013).

In many populations, alcohol is associated with several factors that may influence breast cancer mortality. Female alcohol consumers are more likely to have higher socioeconomic status (Cederfjäll et al. 2004), lower body mass index (BMI) (Sayon-Orea et al. 2011;Li et al. 2010); and higher rates of hormone replacement therapy (HRT) use (Li et al. 2010), antidepressant use (Graham and Massak 2007), and complementary alternative medicine (CAM) use (Hietala et al. 2011). Moreover, increasing alcohol consumption is associated with smoking (Hamajima et al. 2002). Several of these factors may influence hormone levels (Reichman et al. 1993;Ginsburg 1999;Wayne et al. 2008), which may be related to breast cancer mortality (MacMahon et al. 1982;Carmichael 2006;Wurtz et al. 2012). Since lifestyle factors, such as alcohol consumption, are modifiable, more personalized recommendations could be given to the patients based on better knowledge about how these factors may influence breast cancer mortality and recurrence.

The aim of the study was to investigate the association between pre- and postoperative alcohol consumption and risk for early breast cancer events, early distant metastases, and death.

## Materials and methods

### Study population

**Figure 1:**
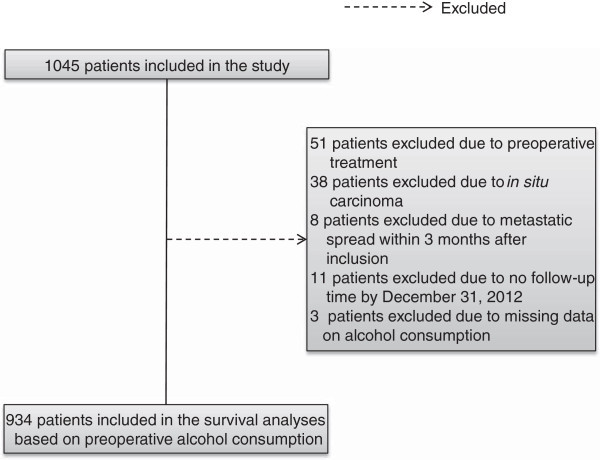
**Flowchart illustrating the inclusion and exclusion criteria for patients in the different analyses.**

This paper is based on data from an on-going population-based cohort study of women with a first diagnosis of breast cancer at Skåne University Hospital, Lund, Sweden. Between October 2002 and December 2011, 1 045 patients were included in the study at the time of diagnosis, and were followed until December 31^st^ 2012. The number of patients included in the analyses in the current study were 934, see Figure 1. Patients diagnosed with another cancer during the last ten years or with a prior history of breast cancer were not included. Previous analyses have shown that patients who were not included were similar to those patients who were included regarding age at diagnosis and ER-status (Lundin et al. 2011;Markkula et al. 2012a). Thus, the patient population is considered to be representative of breast cancer patients in Sweden. The follow-up rates for the patients without preoperative treatment included in the study and who were alive and recurrence-free have previously been shown to be high (Simonsson et al. 2013). The following three types of events were considered: 1) any early breast cancer event including local or regional recurrence, new breast cancer, or distant metastases; 2) distant metastases; 3) death. The primary end-point of the study was any early breast cancer event.

The patients were asked to fill-out questionnaires at a preoperative clinical visit and again after three to six months and one, two, three, five, seven, and nine years postoperatively. Written informed consents were obtained from all patients, and the study was approved by the Ethics Committee of Lund University (LU 75-02, LU 37-08, and LU 658-09). At the preoperative visit anthropometric factors were measured by a trained research nurse. The previously described standardized measurement of breast volume (Ringberg et al. 2006) was utilized for this study. The questionnaires included questions about e.g. reproductive history (Simonsson et al. 2013), use of exogenous hormones, smoking status (Simonsson et al. 2013), coffee consumption (Simonsson et al. 2013;Bågeman et al. 2008), and alcohol consumption. Alcohol consumption was reported in terms of frequency (never, ≤1 time/month, 2-4 times/month, 2-3 times/week, 4+ times/week) at the preoperative visit, and the number of drinks consumed during the past week was reported (none, 1-3 drinks, 4-9 drinks, 10-19 drinks, or 20+ drinks) preoperatively as well as at each follow-up visit. The questions concerning alcohol consumption were obtained from the Alcohol Use Disorders Identification Test (AUDIT) (Saunders et al. 1993).

Information on tumor characteristics (invasive tumor size, axillary lymph node involvement, histological grade, and hormone receptor status) was obtained from each patient’s pathology report. Information on these tumor characteristics was available for 934 (100%), 932 (99.8%), 933 (99.9%), and 932 (99.8%) patients, respectively. The tumors were analyzed at the Department of Pathology at Skåne University Hospital, Lund. ER and PgR status were determined by immunohistochemistry using the Dako LSAB kit system (Dako) and the antibodies M7047 (ER) and M3569 (PR; Dako) until December 2009 (Bågeman et al. 2008;Jernström et al. 2009), thereafter with the antibodies ER (SP1) and PgR (1E2) Ventana Medical Systems’ (Ventana, AZ, USA) on Ventana Benchmark Ultra (Ventana Medical Systems). Tumors with >10% positive nuclear staining were considered ER + or PgR+. Information concerning any breast cancer event, i.e., local or regional recurrence, new breast cancer, and distant metastases, was obtained from patient charts, pathology reports, and the Regional Tumor Registry. Deaths and date of death were obtained from the Swedish Population Registry. Information regarding type of surgery, sentinel node biopsy, axillary node dissection, and type of adjuvant treatment was collected from patient charts. Treatment was administered according to the standard of care at Skåne University Hospital during the time the cohort was compiled. Treatment prior to the last follow-up or prior to any event was recorded.

### Data analyses

Statistical analyses were performed using IBM SPSS statistics 19.0 (Chicago, IL, USA) and Stata (StataCorp. 2013. Stata: Release 12.1. Statistical Software. College Station, TX: StataCorp LP). For analyses of risk for early events and distant metastases, patients were followed from inclusion in the study to the time of the first occurrence of the following events: the first subsequent breast cancer event or distant metastasis, last follow-up, or death prior to January 1^st^, 2013. For all-cause mortality, patients were followed from inclusion to the time of the first occurrence of the following events: last follow-up or death from any cause prior to January 1^st^, 2013.

Alcohol consumption was re-classified into four groups based on reported weekly alcohol consumption: no intake (abstainers, 0 drinks/week), low intake (1-3 drinks/week), moderate intake (4-9 drinks/week), and high intake (10+ drinks/week). Body mass index (BMI) was calculated as weight, (kg)/height, (m)^2^.Intra-individual changes in the amount of alcohol consumption between visits were analyzed with McNemar-Bowker test. To study the association between reported alcohol frequency and intake, a Spearman correlation test was used.

The log-rank test was used to analyze risk for early events in relation to preoperative alcohol consumption. Cox regression was used to calculate crude and adjusted hazard ratios (HR) with 95% confidence intervals (CI) for early events in relation to alcohol consumption. Interaction analyses between any preoperative alcohol consumption and age (50 years or older versus younger), tumor characteristics and breast cancer treatments were performed. Treatment after any breast cancer event was not considered. A series of Cox-regression analyses were performed to study the relationship between current alcohol consumption at any visit (any vs null) and risk of an early breast cancer event. The first analysis examined the period ranging from the preoperative visit onwards, whereas the second analysis excluded the period between the preoperative visit and the first observed postoperative visit time in the cohort. If the patient, at a follow-up visit, reported changing her drinking habits since the previous visit, the dichotomized alcohol consumption variable was updated and the Cox model refitted. The analysis yields an updated HR for current alcohol consumption conditional on being alive and event-free throughout the first time interval. The third model yields a HR conditional on being alive and event-free up to the second postoperative follow-up time in the cohort and so on up to four years of follow-up. If data on current alcohol consumption at the time of a postoperative visit was missing, the value from the previous visit was used in these analyses. The highly dependent estimated HR:s were plotted against follow-up time with 95% point-wise confidence bands. A competing risk analysis was performed using the Stata procedure stcrreg as described by (Fine and Gray (1999)). The effect measures in this model are so-called subhazard ratios (SHR). A *P*-value <0.05 was considered significant. All *P*-values were two-tailed. Nominal *P*-values are presented without adjustments for multiple testing.

## Results

### Patient- and tumor characteristics

Median age at diagnosis was 60.9 years (range 24-99). As presented in Table 1, increasing preoperative alcohol consumption was associated with several lifestyle factors. A shift towards lower alcohol consumption during the 3-6 month visit compared to the preoperative visit was observed, with 25.2% reporting a decreased consumption, 61.2% a stable consumption and the remaining 13.6% an increased consumption, p < 0.0001, McNemar-Bowker test. The tumor characteristics are presented in Table 2. ER + tumors were more common and hormone receptor negative tumors less common among patients with a moderate alcohol intake, compared to abstainers or patients with a high intake.

**Table 1:** **Patient characteristics in a Swedish cohort of 934 patients diagnosed with breast cancer between 2002-2011**

			Preoperative alcohol consumption during the last week				
			Non-consumers	Moderate		High	
	Patients included in the survival analyses	Missing	0 drinks/week	1-3 drinks/week	4-9 drinks/week	10+ drinks/week	
	n = 934		300 (32.1%)	369 (39.5%)	232 (24.8%)	33 (3.5%)	
	Median		Median	Median	Median	Median	
	(IQR) or %		(IQR) or %	(IQR) or %	(IQR) or %	(IQR) or %	
**Age at diagnosis, yrs**	60.6 (52.1-67.6)	0	63.2 (52.8-70.6)	60.9 (52.5-67.7)	59.6 (51.1-65.7)	58.4 (52.9-67.1)	
**Weight, kgs**	69.0 (62.0-78.0)	18	70.0 (60.8-79.1)	68.0 (62.1-78.4)	69.0 (61.0-76.0)	73.0 (60.5-80.0)	
**Height, m**	1.65 (1.62-1.70)	18	1.64 (1.60-1.68)	1.66 (1.62-1.70)	1.67 (1.63-1.70)	1.68 (1.64-1.75)	
**BMI, kgs/m** ^**2**^	24.9 (22.5-28.2)	20	25.8 (23.0-29.3)	24.9 (22.7-28.4)	24.8 (21.9-27.4)	24.4 (22.0-28.4)	
**Waist-Hip Ratio**	0.85 (0.80-0.90)	28	0.87 (0.81-0.91)	0.85 (0.80-0.89)	0.85 (0.80-0.90)	0.83 (0.79-0.89)	
**Total breast volume in patients without previous breast surgery, mL**	1000 (650-1550)	138	1000 (700-1580)	1000 (625-1450)	950 (644-1600)	1000 (600-1650)	
**Age at menarche, yrs**	13.0 (12.0-14.0)	4	13.0 (13.5-14.0)	13.0 (12.0-14.0)	13.0 (12.0-14.0)	13.0 (12.3-14.0)	
**Parous, %**	87.6	1	86.3	87.8	90.1	81.8	
**Age at first full term pregnancy, yrs**	25.0 (22.0-28.0)	122	24.0 (20.0-28.0)	25.0 (22.0-28.0)	25.0 (22.5-28.0)	26.0 (23.0-27.0)	
**Ever use of oral contraceptives, %**	71.6	1	63.5	68.8	81.5	84.8	
**Ever use of HRT, %**	44.2	2	37.8	48.1	47.0	42.4	
**Current smoker prior to surgery, %**	20.9	1	17.4	19.8	23.7	33.3	
**Preoperative daily coffee consumption >2cups/day**	81.9	4	77.3	82.2	81.9	93.9	
**Antidepressants, %**	10.6	5	15.0	6.0	11.3	9.1	
**Preoperative use of Complementary alternative medicine**	23.5	9	22.5	19.9	24.5	40.6	
**Reported frequency of preoperative alcohol consumption**		6					rs = 0.70 (P < 0.0001)
Never	95 (10.2)		95 (31.9)	0 (0.0)	0 (0.0)	0 (0.0)	
≤ 1 time/month	243 (26.2)		148 (49.7)	76 (20.7)	19 (8.3)	0 (0.0)	
2-4 times/month	363 (39.1)		49 (16.4)	224 (61.0)	85 (37.0)	5 (15.2)	
2-3 times/week	174 (18.8)		4 (1.3)	66 (18.0)	89 (38.7)	15 (45.5)	
4+ times/week	53 (5.7)		2 (0.7)	1 (0.3)	37 (16.1)	13 (39.4)

**Table 2:** **Tumor characteristics in a Swedish cohort of 934 patients diagnosed with breast cancer between 2002-2011**

		Preoperative alcohol consumption during the last week			
		Non-consumers	Moderate		High
	Patients included in the survival analyses	0 drinks/week	1-3 drinks/week	4-9 drinks/week	10+ drinks/week
	n = 934	300 (32.1%)	369 (39.5%)	232 (24.8%)	33 (3.5%)
Neoadjuvant therapy	0	0	0	0	0
Preoperative interstitial laser thermotherapy	0	0	0	0	0
Missing	0	0	0	0	0
**No preoperative treatment**	934	272	343	217	32
**Pathological tumor size**					
*In Situ*	0 (0.0%)	0 (0.0%)	0 (0.0%)	0 (0.0%)	0 (0.0%)
1 (≤20 mm)	679 (72.47)	215 (71.7%)	271 (73.4%)	169 (72.8%)	24 (72.7%)
2 (21-50 mm)	238 (25.5%)	77 (25.7%)	92 (24.9%)	60 (25.9%)	9 (27.3%)
3 (≥51 mm)	15 (1.6%)	8 (2.7%)	5 (1.4%)	2 (0.9%)	0 (0.0%)
4 (skin or muscular involvement independent of size)	2 (0.2%)	0 (0.0%)	1 (0.3%)	1 (0.4%)	0 (0.0%)
Invasive tumor size ≥21 mm or skin or muscular involvement	255 (27.3%)	85 (28.3%)	98 (26.6%)	63 (27.2%)	9 (27.3%)
Missing	0	0	0	0	0
**Axillary node involvement**					
0	572 (61.4%)	188 (63.1%)	227 (61.5%)	134 (57.8%)	23 (69.7%)
1-3	277 (29.5%)	80 (26.8%)	111 30.1%)	78 (33.6%)	8 (24.2%)
4+	83 (8.9%)	30 (10.1%)	31 (8.4%)	20 (8.6%)	2 (6.1%)
Any axillary lymph node	360 (38.6%)	110 (36.9%)	142 (38.5%)	98 (42.2%)	10 (30.3%)
Missing	2	2	0	0	0
**Histological grade**					
I	229 (24.5%)	64 (21.4%)	103 (27.9%)	54 (23.3%)	8 (24.2%)
II	472 (50.6%)	158 (52.8%)	175 (47.4%)	118 (50.9%)	21 (63.6%)
III	232 (24.9%)	77 (25.8%)	91 (24.7%)	60 (25.9%)	4 (12.1%)
Missing	1	1	0	0	0
**Hormone receptor status**					
ER+	813 (87.2%)	249 (83.0%)	334 (90.8%)	202 (87.4%)	28 (84.8%)
PgR+	656 (70.4%)	205 (68.3%)	260 (70.7%)	168 (72.7%)	23 (69.7%)
ER + PgR+	650 (69.7%)	202 (67.3%)	259 (70.4%)	166 (71.9%)	23 (69.7%)
ER + PgR-	163 (17.5%)	47 (15.7%)	75 (20.4%)	36 (15.6%)	5 (15.2%)
ER-PgR-	113 (12.1%)	48 (16.0%)	33 (9.0%)	27 (11.7%)	5 (15.2%)
ER-PgR+	6 (0.6%)	3 (1.0%)	1 (0.3%)	2 (0.9%)	0 (0.0%)
Missing	2	0	1	1	0

### Alcohol consumption in relation to early events, distant metastases, and all-cause mortality

**Figure 2:**
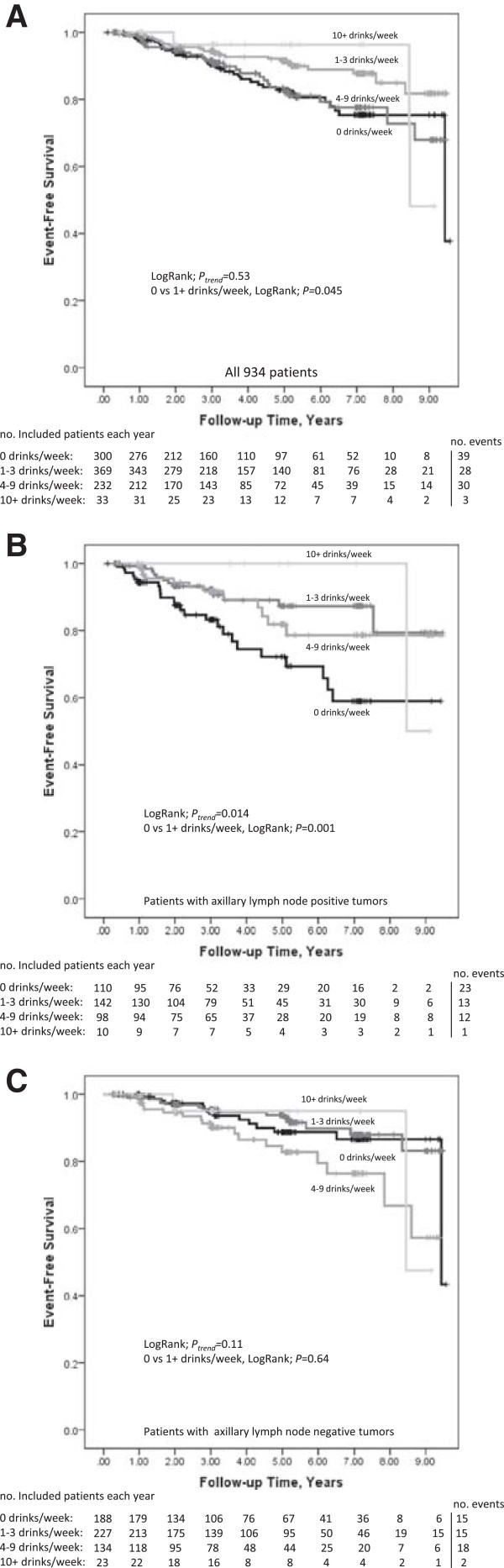
**A-C** **Preoperative alcohol consumption in relation to early events, including local or regional recurrence, new breast cancer, or distant metastases, in:** **A)** All patients – adjusted HR 0.55 (95% CI 0.34-0.91) for 1-3 drinks/week, adjusted HR 0.90 (95% CI 0.55-1.48) for 4-9 drinks/week, and adjusted HR 0.71 (95% CI 0.21-2.32) for 10+ drinks/week. Adjusted HR for any alcohol consumption: 0.69 (95% CI 0.45-1.04). **B)** Patients with axillary lymph node involvement. – adjusted HR 0.38 (95% CI 0.19-0.77) for 1-3 drinks/week, adjusted HR 0.52 (95% CI 0.25-1.07) for 4-9 drinks/week, and adjusted HR 0.33 (95% CI 0.04-2.55) for 10+ drinks/week. There were no events in the group with 10+ drinks/week. Adjusted HR for any alcohol consumption: 0.43 (95% CI 0.24-0.77). **C)** Patients without axillary lymph node involvement – adjusted HR 0.93 (95% CI 0.44-1.94) for 1-3 drinks/week, adjusted HR 1.81 (95% CI 0.87-3.75) for 4-9 drinks/week, and adjusted HR 1.28 (95% CI 0.28-5.82) for 10+ drinks/week. Adjusted HR for any alcohol consumption: 1.24 (95% CI 0.66-2.34). Since this is an on-going cohort, the number of patients decreases as the follow-up time increases.

The median follow-up time until any breast cancer event, the patient’s completion of the last questionnaire, or death was 3.0 years (interquartile range 2.0-5.2). For the total number of events and deaths in each alcohol category and for univariable and multivariable models, see Tables 3. Higher preoperative alcohol consumption was not associated with lower risk for early breast cancer events compared to no consumption (*P*_trend_ = 0.53), Figure 2A and Table 3. Any preoperative alcohol consumption was non-significantly associated with lower risk for early events compared to no consumption, adjusted HR 0.69 (95% CI 0.45-1.04). The analyses were adjusted for age at diagnosis, invasive tumor size, axillary lymph node involvement, tumor grade III, ER status, BMI, preoperative current smoking, and treatments. Any alcohol consumption was also associated with lower risk for early distant metastases, adjusted HR 0.60 (95% CI 0.36-1.00), and death, adjusted HR 0.62 (95% CI 0.38-1.01). Of the 76 patients who died during follow-up, 45 patients had experienced an event. A competing risk regression analysis using the model (Fine and Gray (1999)) led to essentially the same results, adjusted SHR 0.69 (95% CI 0.45-1.05) for 1-9 drinks/week and adjusted SHR 0.71 (95% CI 0.25-2.05) for 10+ drinks/week, compared to null consumption.

**Table 3:** **Pre- and postoperative alcohol consumption in relation to risk for early breast cancer events (significant associations are indicated in bold)**

		Risk for early breast cancer events										
			All		Lymph node negative^c^				Lymph node positive^c^			
Alcohol consumption	No.	No. of events	Crude HR	Adj. HR^a^	No.	No. of events	Crude HR	Adj. HR^b^	No.	No. of events	Crude HR	Adj. HR^b^
			(95% CI)	(95% CI)			(95% CI)	(95% CI)			(95% CI)	(95% CI)
Preoperative	934	100			572	49			360	50		
None	300	39	Ref.	Ref.	188	14	Ref.	Ref.	110	24	Ref.	Ref.
Any (1+ drinks/week)	634	61	**0.66 (0.44-0.99)**	0.69 (0.45-1.04)	384	35	1.16 (0.62-2.15)	1.32 (0.69-2.52)	250	26	**0.40 (0.23-0.70)**	**0.42 (0.23-0.75)**
0 drinks/week	300	39	Ref.	Ref.	188	14	Ref.	Ref.	110	24	Ref.	Ref.
1-3 drinks/week	369	28	**0.51 (0.31-0.83)**	**0.55 (0.34-0.91)**	227	15	0.79 (0.38-1.63)	0.99 (0.47-2.10)	142	13	**0.36 (0.18-0.71)**	**0.37 (0.18-0.74)**
4-9 drinks/week	232	30	0.94 (0.58-1.51)	0.90 (0.55-1.48)	134	18	1.93 (0.96-3.90)	1.89 (0.90-3.96)	98	12	**0.47 (0.23-0.94)**	0.51 (0.25-1.04)
10+ drinks/week	33	3	0.59 (0.18-1.92)	0.70 (0.21-2.32)	23	2	1.13 (0.26-4.98)	1.35 (0.30-6.15)	10	1	0.31 (0.04-2.32)	0.31 (0.04-2.36)

^a^Adjusted for age at diagnosis (linear), invasive tumor size (<21 mm versus ≥21 mm or skin or muscular involvement independent of size), axillary lymph node involvement (yes/no), tumor grade III (yes/no), ER status (+/−), BMI (<25.0 kg/m2), preoperative current smoking, and treatment; tamoxifen, aromatase inhibitors, chemotherapy, and radiation therapy (missing data for 26 patients and 0 patients, respectively).

^b^Adjusted for age at diagnosis, invasive tumor size, tumor grade, ER status, BMI, preoperative current smoking, and treatment; tamoxifen, aromatase inhibitors, chemotherapy, and radiation therapy.

^c^Missing data on lymph node status for 2 patients.

In order to determine whether the association between alcohol consumption and risk for early events differed by age and tumor characteristics, analyses of interaction effects were performed. There were no significant interactions between age at diagnosis (50 years or older), ER-positivity, invasive tumor size (≥21 mm or skin or muscular involvement independent of size), or histological grade III and any preoperative alcohol consumption (*P* > 0.1). However, there was a significant interaction between any alcohol consumption and any axillary lymph node involvement (adjusted HR 2.94 95% CI 1.26-6., adjusted *P*_interaction_ = 0.01). Therefore, exploratory studies stratified according to axillary lymph node involvement were performed. Any alcohol consumption was significantly associated with lower risk for early events among patients with axillary lymph node involvement, Figure 2B, adjusted HR 0.42 (95% CI; 0.23-0.75) but not in the lymph node negative patients, Figure 2C, adjusted HR 1.32 (95% CI; 0.69-2.52).

Among the 813 patients with ER + tumors, 480 patients received tamoxifen and 307 received AI. Out of the 943 patients included in the survival analyses, 238 patients received chemotherapy and 587 patients received radiation therapy. Interaction analyses by treatment type and any preoperative alcohol consumption were performed, but none were significant (*P* > 0.05). Further adjustments for potential confounders (preoperative waist to hip ratio, previous treatment for menopausal symptoms, preoperative coffee consumption, preoperative CAM use, and preoperative antidepressant use) did not materially change the results (data not shown).

### Current alcohol consumption in relation to early events

**Figure 3:**
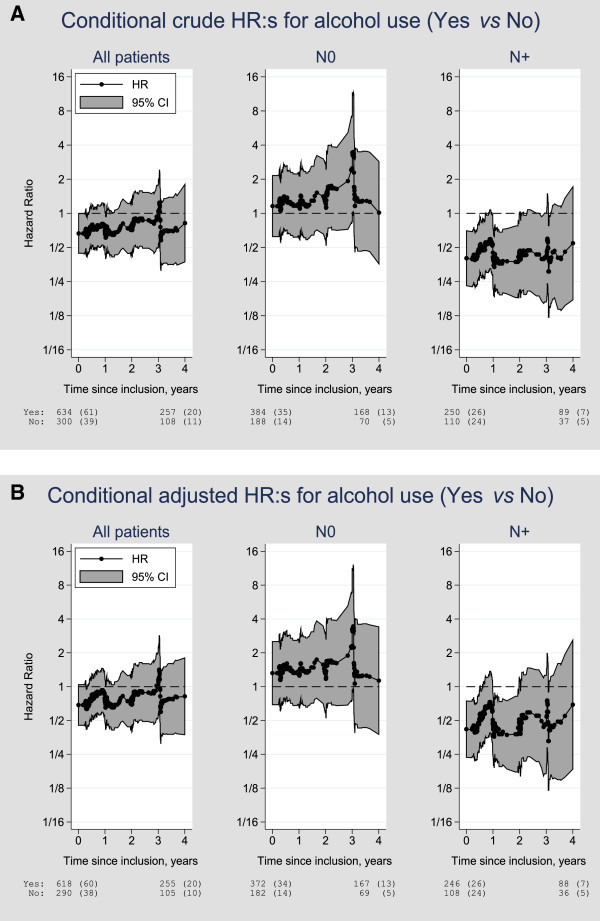
**A-B Unadjusted and adjusted conditional HR:s for alcohol use.** The figures show estimated hazard ratios (HR), with 95% point wise confidence intervals (CI), for alcohol use (yes vs no) from a series of Cox-regression analyses of time from the preoperative visit to any breast cancer event. Each dot corresponds to an analysis conditional on event-free survival up to the corresponding observed visit time. Crude (unadjusted) estimates are shown in Figure 3A and adjusted estimates in Figure 3B. The panels show, from left to right, analyses for all patients, axillary lymph node negative (N0), and axillary lymph node positive (N+) patients, respectively. The number of patients at risk in each of the two groups at time of diagnosis and four years post-operatively is shown below each panel, and the corresponding number of events observed is shown between parentheses. The reason why the counts in N0 and N + in Figure 3A do not sum to the counts for all patients in the group who report no alcohol use is that the axillary lymph node status is unknown for two patients.

The results presented above refer to the association between preoperative alcohol use, i.e. a characteristic known at the time of diagnosis, and prognosis. Updated information on alcohol use at each subsequent visit was related to prognosis by means of a series of Cox-regression analyses conditional on event-free survival up to time *t*, where *t* varies from 0 to 4 years of follow-up. The method is described in more detail in the section Data Analyses. The results of these analyses are summarized in Figure 3A, univariable, and Figure 3B, multivariable analyses. Briefly, the analyses indicate that any alcohol use at the time of diagnosis was weakly associated with a lower risk of early breast cancer events (HR < 1.0). For all patients (left panels), this association leveled off with time and it was non-significant at the 5% level, but the point estimate of the HR for any current alcohol consumption was below 1.0 up to 3 years after diagnosis. The two panels to the right in the figures show the estimated conditional HR:s by axillary lymph node status. There was no association between alcohol consumption at any point during follow-up in patients with axillary lymph node negative tumors. However, for patients with axillary lymph node positive tumors a moderate association was seen between any alcohol intake and a lower risk for early events.

## Discussion

The present study investigated the association between alcohol consumption and risk for early breast cancer events in an ongoing cohort of primary breast cancer patients. The main result was that any preoperative alcohol consumption was weakly associated with lower risk for early breast cancer events and early distant metastases. Using updated information on alcohol use at each subsequent visit postoperatively by means of a series of Cox-regression analyses also indicated a weak association between any alcohol consumption and lower risk for early events with point estimates of the HR for any current alcohol consumption below 1.0 up to 3 years after diagnosis. Secondly, as a result of exploratory subgroup analyses, any alcohol consumption was associated with lower risk for early events among patients with axillary lymph node involvement.

The availability of data on alcohol consumption during repeated time points during the follow-up period is a strength of this study. Assessing alcohol consumption shortly after surgery (which coincides with chemotherapy or radiation therapy in many patients) may yield too low estimates of alcohol consumption. Additionally, self-reported alcohol consumption is often underestimated (Embree and Whitehead 1993) and may constitute a self-presentational bias. Consistent with this hypothesis, nearly half of the patients who claimed to never consume alcohol at the preoperative visit reported consuming alcohol during at least one follow-up visit, in the present study. The preoperative alcohol consumption in this study is therefore likely to be underreported. Underreporting would have biased the results towards the null hypothesis. However, with updated information of postoperative alcohol consumption in this study, the results remained.

Several possible mechanisms for the effect of alcohol in carcinogenesis have been proposed, but the mechanisms for how alcohol affects the tumor after diagnosis has not been sufficiently studied. Vorderstrasse *et al*. reported in a mouse model study with mammary tumor transplantation, that alcohol consumption, especially in high but also in moderate doses, protected against distant metastases compared with water consumption (Vorderstrasse et al. 2012). One hypothesis might be that while alcohol consumption confers a higher risk for breast cancer, it gives rise to tumors with better prognosis, a phenomenon reported for HRT (Jernström et al. 1999;Jernström et al. 2003). In the present study, preoperative moderate alcohol consumption was associated with a lower frequency of ER–PgR– tumors and a higher frequency of ER + tumors, but was associated with a better prognosis irrespective of ER-status. Moreover, an increase in the plasma ratio of the estrogen metabolites 2OHE to 16αOHE with increased alcohol consumption has been shown in a pilot study of the present cohort (Klug et al. 2006). This may lead to lower stimulation of the ER since 2OHE acts as weak or anti-estrogen and the 16αOHE1 has been shown to be procarcinogenic (Schneider et al. 1984). Additionally, in a subset of the current cohort, seldom drinkers (<2 times/month) were found to be less adherent to endocrine therapy (Markkula et al. 2012b), which may be another explanation for the increased risk for early events in patients with ER + tumors, but it does not explain an increased risk in patients with axillary lymph node involvement. The difference in association between any alcohol consumption and early events between patients with and without axillary lymph node involvement warrants further investigation. The subgroup analysis was not pre-specified and no adjustments for multiple testing were performed. One biologically plausible hypothesis might be that any remaining tumor cells from tumors that had already metastasized to the axillary lymph nodes at the time of diagnosis differed with respect to response to alcohol from those that had metastasized to other sites or not metastasized at the time of diagnosis. An alternative hypothesis is that the difference can be attributed to the higher frequency of early events in patients with axillary lymph node involvement compared to patients without axillary lymph node involvement. These patients may therefore be more likely to be detected in this study. Since this study has a short median follow-up time, the results warrant confirmation in a study with longer follow-up.

The weak association between any alcohol consumption and a lower risk for early events found in the present study is in contrast to the results of five previous studies (Kwan et al. 2010;Holm et al. 2013;Flatt et al. 2010;Vrieling et al. 2012;Kwan et al. 2013). Several explanations could account for the different results from previous studies, for example the use of different alcohol variables, such as preoperative alcohol consumption and consumption during follow-up. Another reason for inconsistent results from previous studies may be that different endpoints have been used, such as overall survival (Holmes et al. 1999;Dal Maso et al. 2008;Hellmann et al. 2010;Kwan et al. 2010;Harris et al. 2012;Reding et al. 2008;Barnett et al. 2008;Flatt et al. 2010), breast cancer specific survival (Dal Maso et al. 2008;Kwan et al. 2010;Harris et al. 2012;Holm et al. 2013;Reding et al. 2008;Flatt et al. 2010;Allemani et al. 2011;Vrieling et al. 2012), and breast cancer recurrence (Kwan et al. 2010;Holm et al. 2013;Flatt et al. 2010;Vrieling et al. 2012;Kwan et al. 2013). Furthermore, no international consensus has been established to define a standard drink or low-risk alcohol consumption (Furtwaengler and de Visser 2013). According to the Swedish recommendations, a standard drink is considered to consist of 12 grams of ethanol (Furtwaengler and de Visser 2013), which equals approximately one glass of wine or 4 cl of 40% liquor. The maximum recommended alcohol consumption for women in Sweden is 108 g/week, which equals nine drinks/week (Furtwaengler and de Visser 2013). This correlates with the classifications made in this study (10+ drinks/week classified as high intake). The size of the glasses and the type of beverage were not specified in the present study. However, a Swedish cohort of over 10,000 women reported similar median alcohol intake as the present study (Cederfjäll et al. 2004). Additionally, the questionnaire did not include questions about the patients’ consumption in between visits. The preoperative frequency of alcohol consumption was therefore also examined in relation to the risk for early events and distant metastases. The association between higher frequency of alcohol consumption and both types of events remained significant among patients with axillary lymph node involvement (data not shown).

Higher socioeconomic status has been associated with increased breast cancer-free survival (Halmin et al. 2008), increased alcohol intake (Cederfjäll et al. 2004), and decreased levels of smoking (MacMahon et al. 1982). Conversely, increasing alcohol intake is associated with smoking (Hamajima et al. 2002). For the present study, data on socioeconomic status was not available and could constitute a potential confounder. Low alcohol consumption, especially in combination with smoking, could thus be a marker for lower socioeconomic status. However, according to a recent study, the association between educational status and breast cancer-free survival might actually be explained by co-morbidities and lifestyle factors such as alcohol consumption (Aarts et al. 2012). A weakness of the present study is that the questionnaire did not include questions concerning history of alcohol consumption or co-morbidities. Some of the non-consumers in the current cohort may have other co-morbidities that may influence drinking behaviors, morbidity, and mortality. Moreover, some of the abstainers in this study could be former alcohol abusers. Lifelong abstainers and former drinkers have been reported to have higher morbidity and mortality rates than light or moderate drinkers (Green and Polen 2001).

Lower reported alcohol consumption was observed during the 3-6 months visit compared to the pre- or postoperative consumption. This may be due to nausea or altered taste in chemotherapy treated patients. The lower alcohol consumption at the 3-6 months visit compared to the pre-operative consumption is indirectly shown graphically in Figures 3A and B as conditional HRs well below 1.0 pre-operatively approaching the dashed line HR = 1.0 as the starting time of the analyses increases up to 1 year. This decreasing effect was mainly driven by patients switching from the group with best prognosis (alcohol consumers) to the reference group (non-consumers) during this time period. The extreme fluctuation of the estimates around three years post-operatively results mainly from the fact that the 3-year visit is the last visit recorded for a large fraction of the patients in the cohort. Hence, the remaining cohort of patients who contribute to the estimated effects in the time window from three to four years is considerably smaller, which is also reflected in the wider 95% CIs.

This study is based on a prospective cohort of breast cancer patients, a strength of the study, because retrospective studies tend to miss patients with early events. Skåne University Hospital in Lund has a catchment area of nearly 300,000 inhabitants. Since patients with breast cancer diagnoses are not referred to other hospitals for surgery, this study population is considered population based.

## Conclusions

Alcohol consumption, preoperative as well as postoperative, seems to be weakly associated with fewer early breast cancer events and fewer distant metastases, but not with all-cause mortality. Patients have the possibility of modifying their drinking habits, and the results of this study do not support recommending all breast cancer patients to abstain from low to moderate alcohol consumption. These results warrant confirmation in studies with longer follow-up.

## Funding

This work was supported by grants from The Swedish Cancer Society CAN 2011/497, the Swedish Research Council K2012-54X-22027-01-3 (PI H Jernström), the Medical Faculty at Lund University, the Mrs. Berta Kamprad Foundation, the Gunnar Nilsson Foundation, the Swedish Breast Cancer Group (BRO), the South Swedish Health Care Region (Region Skåne ALF), Konung Gustaf V:s Jubileumsfond, and the Lund Hospital Fund.
